# Supplementary material for: A Screening Measure of Emotion Regulation Difficulties: Polish Norms and Psychometrics of the Difficulties in Emotion Regulation Scale-8 (DERS-8)
Source: Healthcare (Basel). 2025 Feb 18;13(4):432. doi: 10.3390/healthcare13040432 (PMC11855059; doi:10.3390/healthcare13040432)
Supplement: Supplementary file 1 [file healthcare-13-00432-s001.zip › healthcare-3439845-supplementary.pdf]

Supplementary Table S1. Percentile ranks norms for DERS-8 scores in the total sample (n = 1329)

Raw Score	Percentile Rank	95% Credible Interval		90% Credible Interval	
		Lower	Upper	Lower	Upper
8	0.6	0.0	1.5	0.0	1.3
9	1.6	0.8	2.6	0.9	2.4
10	2.6	1.6	3.9	1.8	3.7
11	4.7	2.9	6.7	3.1	6.4
12	7	5.5	9.4	5.7	9.1
13	10	7.9	12.5	8.2	12.1
14	14	10.9	16.9	11.3	16.5
15	18	15.0	20.5	15.4	20.0
16	21	18.3	24.6	18.7	24.2
17	26	22.4	28.9	22.8	28.4
18	30	26.5	33.1	26.9	32.6
19	34	30.4	37.2	30.9	36.6
20	38	34.5	42.3	35.1	41.7
21	43	39.4	45.9	39.9	45.4
22	47	42.9	50.1	43.4	49.6
23	51	47.3	54.5	47.8	54.0
24	55	51.7	58.9	52.2	58.4
25	60	56.1	63.1	56.6	62.6
26	64	60.4	68.1	61.0	67.5
27	69	65.6	72.8	66.2	72.3
28	73	70.1	75.8	70.6	75.4
29	76	73.0	78.6	73.4	78.2
30	79	76.0	81.7	76.4	81.3
31	82	79.4	85.3	79.9	84.9
32	86	83.4	88.7	83.8	88.4
33	89	86.9	91.5	87.3	91.2
34	92	89.8	93.7	90.1	93.4
35	94	92.2	95.8	92.5	95.6
36	96.0	94.5	97.3	94.8	97.1
37	97.3	96.2	98.3	96.4	98.1
38	98.2	97.2	98.9	97.4	98.8
39	98.7	97.9	99.3	98.1	99.2
40	99.5	98.6	100.0	98.8	100.0

Supplementary Table S2. Percentile ranks norms for DERS-8 scores in the younger adult sample aged 18–29 (*n* = 900)

Raw Score	Percentile Rank	95% Credible Interval		90% Credible Interval	
		Lower	Upper	Lower	Upper
8	0.2	0.0	0.7	0.0	0.6
9	0.6	0.2	1.3	0.2	1.2
10	1.1	0.5	2.0	0.5	1.8
11	2.3	1.0	3.9	1.2	3.7
12	3.9	2.6	5.6	2.8	5.4
13	6	3.9	7.6	4.2	7.3
14	8	5.9	11.3	6.2	10.8
15	12	9.1	14.2	9.4	13.8
16	15	11.7	17.9	12.1	17.4
17	19	15.3	22.2	15.8	21.7
18	23	19.4	26.7	19.9	26.1
19	27	23.4	30.5	24.0	29.9
20	31	27.1	35.3	27.7	34.7
21	35	31.7	39.0	32.3	38.5
22	39	35.2	43.0	35.7	42.4
23	44	39.4	47.8	40.0	47.2
24	48	44.1	52.2	44.7	51.5
25	53	48.4	56.8	49.0	56.2
26	58	53.4	62.8	54.1	62.2
27	64	59.6	68.1	60.2	67.4
28	68	64.3	71.2	64.9	70.7
29	71	67.1	73.8	67.7	73.3
30	74	70.1	77.1	70.6	76.6
31	78	74.0	81.6	74.5	81.1
32	83	79.0	86.0	79.5	85.5
33	87	83.6	89.6	84.0	89.2
34	90	87.3	92.4	87.7	92.1
35	93	90.4	95.1	90.8	94.8
36	95.4	93.5	97.0	93.8	96.8
37	96.9	95.4	98.0	95.7	97.9
38	97.9	96.7	98.9	96.9	98.8
39	98.7	97.7	99.3	97.9	99.2
40	99.4	98.4	100.0	98.6	100.0

Supplementary Table S3. Percentile ranks norms for DERS-8 scores in the older adult sample aged 30–73 (*n* = 429)

Raw Score	Percentile Rank	95% Credible Interval		90% Credible Interval	
		Lower	Upper	Lower	Upper
8	1.4	0.0	3.8	0.1	3.4
9	3.7	1.9	6.2	2.2	5.8
10	6	3.6	9.0	3.9	8.5
11	10	6.1	13.9	6.6	13.3
12	14	10.5	19.0	11.0	18.3
13	19	14.9	24.5	15.5	23.7
14	25	20.0	30.7	20.8	29.9
15	31	25.6	35.8	26.3	35.0
16	35	30.1	40.9	31.0	40.1
17	40	34.9	45.4	35.7	44.6
18	44	38.9	49.1	39.7	48.3
19	48	42.6	53.6	43.5	52.8
20	53	47.7	59.2	48.6	58.4
21	58	52.9	63.0	53.8	62.2
22	62	56.6	67.4	57.5	66.6
23	66	61.3	71.1	62.1	70.4
24	70	65.2	75.3	66.0	74.6
25	74	69.6	78.6	70.3	77.9
26	77	72.7	81.3	73.4	80.6
27	81	75.9	84.7	76.7	84.1
28	84	79.8	87.5	80.5	87.0
29	87	82.9	90.4	83.6	89.9
30	90	86.3	92.8	86.9	92.4
31	92	89.0	94.6	89.5	94.2
32	94	90.8	95.7	91.3	95.4
33	95	92.2	96.7	92.6	96.4
34	95.8	93.5	97.5	93.9	97.3
35	96.6	94.5	98.1	94.9	97.9
36	97.4	95.5	98.7	95.8	98.6
37	98.3	96.6	99.3	96.9	99.1
38	98.6	97.1	99.4	97.4	99.3
39	98.8	97.4	99.6	97.7	99.5
40	99.5	98.2	100.0	98.5	100.0

The Polish version of the Difficulties in Emotion Regulation Scale–8 (DERS–8)

Skala trudności w regulacji emocji–8

Instrukcja. Proszę wskaż, jak często poniższe stwierdzenia odnoszą się do Ciebie, zaznaczając odpowiednią liczbę z poniższej skali obok każdego stwierdzenia.

	Stwierdzenia	Prawie nigdy (0–10%)	Czasami (11–35%)	Około połowę czasu (36–65%)	Większość czasu (66–90%)	Prawie zawsze (91–100%)
1	Kiedy jestem zaniepokojony/a, mam trudności z wykonywaniem pracy lub zadania.	1	2	3	4	5
2	Kiedy jestem zaniepokojony/a, czuję, że nie mam nad sobą kontroli.	1	2	3	4	5
3	Kiedy jestem zaniepokojony/a, wstydzę się, że tak się czuję.	1	2	3	4	5
4	Kiedy jestem zaniepokojony/a, mam trudności z kontrolowaniem mojego zachowania.	1	2	3	4	5
5	Kiedy jestem zaniepokojony/a, uważam, że nic nie mogę zrobić, żeby poczuć się lepiej.	1	2	3	4	5
6	Kiedy jestem zaniepokojony/a, irytuje mnie to, że tak się czuję.	1	2	3	4	5
7	Kiedy jestem zaniepokojony/a, jest mi trudno myśleć o czymkolwiek innym.	1	2	3	4	5
8	Kiedy jestem zaniepokojony/a, dużo czasu zajmuje mi, żeby poczuć się lepiej.	1	2	3	4	5

Skala trudności w regulacji emocji (*Difficulties in Emotion Regulation Scale–8*; DERS–8) to 8-pozycyjny kwestionariusz samoopisowy oceniający poziom trudności w regulacji emocji. Aby obliczyć wynik DERS–8, należy zsumować wszystkie odpowiedzi.
